# Increased circulating total bile acid levels were associated with organ failure in patients with acute pancreatitis

**DOI:** 10.1186/s12876-020-01243-w

**Published:** 2020-07-13

**Authors:** Xiaochun Xie, Jie Dong, Guotao Lu, Kun Gao, Xiaoyao Li, Wenjian Mao, Faxi Chen, Zhihui Tong, Baiqiang Li, Weiqin Li

**Affiliations:** 1 Surgical Intensive Care Unit (SICU), Department of General Surgery, Jinling Hospital, Nanjing Medical University, Nanjing, 210002 People’s Republic of China; 2 Pancreatic Center, Department of Gastroenterology, Affiliated Hospital of Yangzhou University, Yangzhou University, Yangzhou, 225000 People’s Republic of China; 3 Surgical Intensive Care Unit (SICU), Department of General Surgery, Jinling Hospital, Medical School of Nanjing University, Nanjing, 210002 People’s Republic of China; 4 Surgical Intensive Care Unit (SICU), Department of General Surgery, Jinling Hospital, South Medical University, Nanjing, 210002 People’s Republic of China

**Keywords:** Total bile acid, Acute pancreatitis, Organ failure, Retrospective study, Pancreatic necrosis

## Abstract

**Background:**

Recent studies have shown that bile acids (BAs) are closely related to metabolic and inflammatory diseases. Our study aimed to investigate whether circulating total bile acid (TBA) levels were associated with the severity of acute pancreatitis (AP).

**Methods:**

We retrospectively collected data on patients diagnosed with AP in a tertiary center from 01 January 2014 to 31 December 2016. The highest TBA value during the first 1,2,3,5,7 days after admission was determined as D1, D2, D3, D5, D7 TBA_max_. Patients were divided into the high TBA (HTBA) group and the normal TBA (NTBA) group according to whether the TBA_max_ was ≥10 μmol/L. The prognosis and complications, including death, organ failure (OF) and pancreatic necrosis, were compared between the two groups. Logistic regression analysis and receiving operating characteristic (ROC) curve were used to evaluate the relationship between circulating TBA and organ failure in AP patients.

**Results:**

Through stratified analysis of each time period, we found that the incidence of OF in the HTBA group was significantly higher than that in the NTBA group, and the AP severity classification in the HTBA group was more serious than that in the NTBA group. In addition, according to the D7 TBA_max_ values, the pancreatic necrosis rate, percutaneous catheter drainage (PCD) rate and mortality in the HTBA group were higher than those in the NTBA group. Multivariate regression analysis showed that HTBA (odds ratio (OR), 4.894; *P* = 0.002) was an independent risk factor for AP complicated with OF, which was verified in the grouping based on D7 TBA_max_. ROC analysis revealed that a circulating D7 TBA_max_ cutoff point of 6.450 umol/L had optimal predictive value for the development of OF in AP patients with an area under the curve of the ROC curve (AUCROC) of 0.777.

**Conclusions:**

The increase of circulating TBA in early stage of AP is independently related to organ failure, which indicates the adverse prognosis of AP patients.

## Background

Acute pancreatitis refers to an acute inflammation occurred in the pancreas, caused by premature activation of the zymogen, the annual incidence rate of which is about 13–45 cases / 100,000 worldwide and it is one of the most common digestive diseases requiring hospitalization [1, 2]. About 15–20% of AP patients whose inflammation is not limited to the pancreas, but also involving the peripancreatic tissue and other distant organs, secondary to the local or systemic complications, developed into severe acute pancreatitis (SAP) [3, 4]. The mortality of SAP is reported as high as 40–70% [5]. The most common systemic complication is organ failure including acute respiratory distress syndrome (ARDS), acute kidney injury (AKI) and shock [6]. Therefore, it is important to identify the risk factors for OF, so that severe patients can be recognized in the early course of AP and receive appropriate and effective interventions.

Bile acids (BAs) are steroidal molecules generated in the liver by cholesterol oxidation, which have multiple physiological functions including stimulation of bile flow, intestinal absorption of lipophilic nutrients, maintenance of cholesterol homeostasis and regulation the metabolism of lipid, glucose and energy [7–9]. In addition, some studies have demonstrated that BAs can also regulate the inflammatory response of organs [9–11] and alleviate endoplasmic stress [10, 12, 13] through dedicated BAs receptors such as the farnesoid X receptor (FXR) and the G-protein coupled receptor TGR5. Dysregulation of BAs transport and impaired BAs receptor signalling may contribute to the pathogenesis of some metabolic diseases such as non-alcoholic fatty liver disease, obesity, type 2 diabetes, and atherosclerosis [14]. The circulating TBA levels are maintained within a certain range under physiological conditions, generally 2–10 μmol/L [15], but in some states of diseases, the levels of circulating TBA will exceed the threshold. An increase in circulating TBA is predominately detected in several hepatobiliary diseases. For example, portosystemic shunting or damaged hepatocytes which are unable to extract the bile acids from the portal blood and extrahepatic obstruction in which bile acids leak directly from the liver to the systemic circulation may contribute to the elevated circulating TBA. Thus, circulating TBA is often used as an effective biomarker for the diagnosis of hepatobiliary diseases [16]. Additionally, some studies illuminated that the levels of circulating TBA were elevated in patients with metabolic diseases such as obesity and type 2 diabetes [17–20].

The studies about the relationship between BAs and AP are currently limited to the damage and inflammation of pancreatic acinar cells in experimental acute pancreatitis induced by retrograde injection of BA into biliopancreatic duct. There have been few clinical studies on the correlation between circulating TBA and AP so far. Maleszka et al. found that the circulating TBA on the first day of AP in patients with biliary etiology was significantly higher compared to those with alcoholic and other etiologies. Therefore, the authors indicated that circulating TBA can be used as an aid to the diagnosis of AP etiology [15]. In this retrospective study, we analysed clinical data of AP patients in a tertiary referral center. The results showed that elevated levels of circulating TBA in the early stages of AP were closely related to the development of organ failure.

## Methods

### Study design and data collecting

This study was a retrospective cohort study of AP patients admitted to the Acute Pancreatitis Treatment Center of Jinling Hospital from 01 January 2014 to 31 December 2016. The study was approved by the ethics committee of the Jinling Hospital, Medical School of Nanjing University. The diagnosis of AP was based on at least two of the following three criteria: (i) abdominal pain suggesting AP, (ii) elevated serum amylase and / or lipase> 3 times the upper limit of normal, and (iii) characteristic AP Computed tomography (CT) findings. We included patients who met the following criteria: (1) within 7 days after onset of AP; (2) TBA values available within 7 days after admission; (3) 18 years ≤ age ≤ 75 years; (4) exclusion of tumor, pregnancy pancreatitis; (5) no renal replacement therapy (RRT) before admission; (6) without ursodeoxycholic acid (UDCA) treatment; (7) without portosystemic shunting or liver disease affecting TBA, such as cirrhosis, primary biliary cholangitis (PBC), primary sclerosing cholangitis (PSC), etc.

In this study, the circulating TBA values were determined by the enzymatic cycling assay, and the results were directly read by the Hitachi 7600 automatic biochemical analyzer. We collected circulating TBA values on different days within 1 week to observe sequential changes in this indicator after admission. The highest TBA value during the first 1,2,3,5,7 days after admission was determined as D1, D2, D3, D5, D7 TBA_max_. In addition, other laboratory test results on the same day as D7 TBA_max_ were also collected for further analysis. And then we divided all patients into the HTBA group and the NTBA group according to whether the circulating TBA_max_ value was ≥10 μmol/L. All data on patients were collected from the database of Pancreatitis Treatment Center including demographics, etiologies, comorbidities, laboratory test results, diagnosis, and clinical outcomes.

### Study outcomes

The primary outcome was organ failure, which is a very important factor that have a causal association with the severity of AP patients. In our study, organ failure was defined for 3 organ systems (cardiovascular, renal, and respiratory) on the basis of the worst measurement over a 24-h period. In patients without pre-existing organ dysfunction, organ failure was defined as either a score of 2 or more in the assessed organ system using the SOFA (Sepsis-related Organ Failure Assessment) score or when the relevant threshold was breached, as shown: 1. (Shock) Cardiovascular: need for inotropic agent; 2. (AKI) Renal: creatinine ≥171 μmol/L (≥2.0 mg/dL); 3. (ARDS) Respiratory: PaO2/FiO2 ≤ 300 mmHg (40 kPa). Persistent organ failure is the evidence of organ failure in the same organ system for 48 h or more, while transient organ failure is less than 48 h [21].

The secondary outcomes included AP classification, pancreatic or peripancreatic necrosis, percutaneous catheter drainage, laparotomy and death. AP classification was divided into mild, moderate, severe and critical according to the DBC classification [21]. Mild AP is characterized by the absence of both (peri)pancreatic necrosis and organ failure, whereas moderate AP is defined by the presence of sterile (peri)pancreatic necrosis and/or transient organ failure. Severe AP refers to the existence of either infected (peri)pancreatic necrosis or persistent organ failure. Finally, patients with critical AP are those who have both infected (peri)pancreatic necrosis and persistent organ failure.

### Statistical analysis

Data involving demographics, AP etiologies, comorbidities, smoking and drinking, and clinical outcomes were compared between patients in the HTBA and the NTBA groups. The categorical variables were described using frequency and percentage. Continuous variables were described using mean ± standard deviation (SD) or median ± interquartile range (IQR), depending on the distribution of the variables. We used the t test to compare the continuous variables of the normal distribution, and the Wilcoxon signed-rank tests compare the non-normally distributed variables. For categorical variables, a chi-square test or Fisher exact test was used.

Univariate logistic regression was used to explore the potential association between several factors and OF. Then, we employed multivariate logistic regression model to determine whether elevated circulating TBA values were independently associated with OF. The multivariate logistic regression model was adjusted for potential confounders (*P* < 0.1 in univariate analysis) and several related variables.

Area Under the Curve of the Receiving Operating Characteristic Curve (AUCROC) analysis was used to define the optimal cutoff point of some important factors to predict the development of OF. All analyses were performed using SPSS, version 20 (SPSS, Chicago, IL, USA). A bilateral *p*-value less than 0.05 was considered to be a statistically significant.

## Results

### Patient demographics and clinical characteristics

**Fig. 1:**
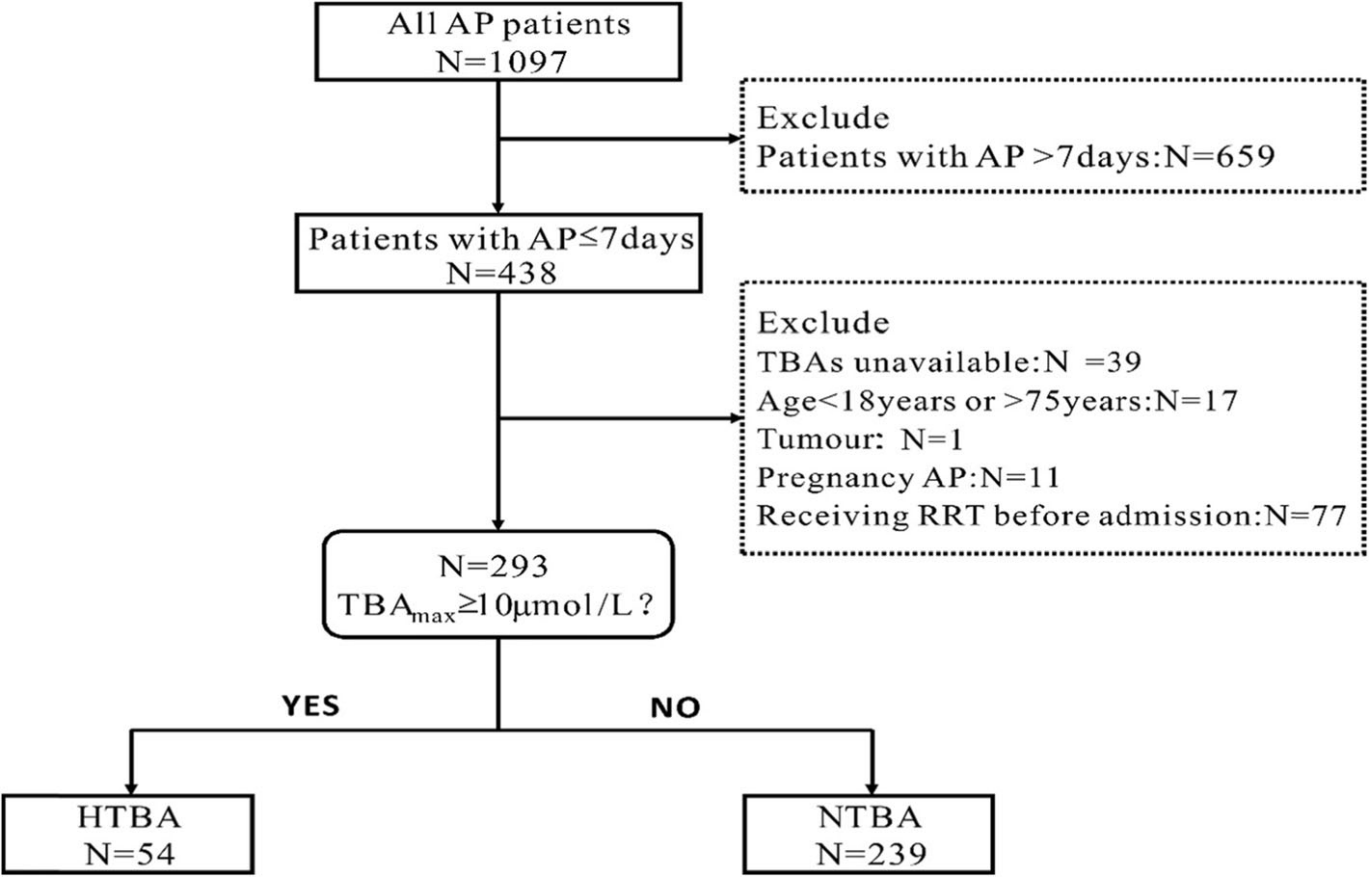
Flow chart of the patients with AP in the study. AP, acute pancreatitis; TBA, total bile acid; RRT, renal replacement therapy; TBA_max_, the highest TBA value within 7 days after admission; HTBA, the high TBA group; NTBA, the normal TBA group

In our study, 1097 AP patients admitted to the Pancreatitis Treatment Center of Jinling Hospital from 01 January 2014 to 31 December 2016 were screened. As show in Fig. 1, a total of 293 patients were eligible for further study (Fig. 1). Demographics and baseline characteristics of patients between the HTBA group and the NTBA group are presented in Table 1. According to different time points, we separately calculated the rate of elevated TBA_max_. As shown in Table 1, from the first day to the seventh day after admission, the rate of elevated TBA_max_ increased from 6.09 to 18.43%. Through stratified analysis of each time period, these patients in the HTBA group were more commonly male when compared with patients in the NTBA group. Notably, patients with HTBA were more likely to be drinking. Whereas, there was no significant differences observed in demographics including age and the etiology of AP between the HTBA group and the NTBA group. Similarly, comorbidities including hypertension, diabetes mellitus (DM), biliary tract disease and fatty liver between both groups did not differ (Table 1).

**Table 1:** Comparison of demographics and clinical characteristics between the HTBA group and the NTBA group

	**D1**			**D2**			**D3**		
**Variable**	**NTBA**	**HTBA**	***P*value**	**NTBA**	**HTBA**	***P*value**	**NTBA**	**HTBA**	***P*value**
	***n*= 262**	***n*= 17**		***n*= 269**	***n*= 22**		***n*= 262**	***n*=31**	
Demographics									
Age, yr, mean±SD	46.7±12.6	48.5±12.2	0.56	46.9±12.5	48.0±13.4	0.699	46.8±12.5	48.5±13.0	0.469
Male, n(%)	162(61.8)	15(88.2)	0.028	169(62.8)	20(90.9)	0.008	163(62.2)	28(90.3)	0.002
BMI, n(%)			0.026			0.035			0.264
<28	173(77.9)	8(50.0)		179(78.5)	11(55.0)		171(77.4)	19(67.9)	
≥28	49(22.1)	8(50.0)		49(21.5)	9(45.0)		50(22.6)	9(32.1)	
Etiology of SAP, n(%)			0.306			0.327			0.357
Biliary	126(48.1)	12(70.6)		131(48.7)	15(68.2)		127(48.5)	20(64.5)	
Hyperlipidemia	110(42.0)	4(23.5)		111(41.3)	6(27.3)		110(42.0)	8(25.8)	
Alcoholic	10(3.8)	0(0)		10(3.7)	0(0)		9(3.4)	1(3.2)	
Idiopathic	16(6.1)	1(5.9)		17(6.3)	1(4.5)		16(6.1)	2(6.5)	
Comorbidities, n(%)									
Hypertension	70(26.7)	8(47.1)	0.125	74(27.5)	9(40.9)	0.181	70(26.7)	13(41.9)	0.075
DM	64(24.4)	3(17.6)	0.733	66(24.5)	3(13.6)	0.248	63(24.0)	6(19.4)	0.561
Biliary tract disease	129(49.2)	10(58.8)	0.444	133(49.4)	11(50.0)	0.96	131(50.0)	14(45.2)	0.61
Fatty liver	115(43.9)	6(35.3)	0.488	118(43.9)	9(40.9)	0.788	117(44.7)	12(38.7)	0.528
Smoking, n(%)	106(40.5)	9(52.9)	0.311	111(41.3)	12(54.5)	0.225	106(40.5)	18(58.1)	0.061
Drinking, n(%)	83(31.7)	8(47.1)	0.19	88(32.7)	12(54.5)	0.038	83(31.7)	18(58.1)	0.003
	**D5**			**D7**					
**Variable**	**NTBA**	**HTBA**	***P*value**	**NTBA**	**HTBA**	***P*value**			
	***n*= 251**	***n*= 42**		***n*= 239**	***n*= 54**				
Demographics									
Age, yr, mean±SD	46.9±12.7	47.3±11.6	0.865	46.7±12.6	48.3±12.2	0.382			
Male, n(%)	153(61.0)	38(90.5)	<0.001	143(59.8)	48(88.9)	<0.001			
BMI, n(%)			0.098			0.141			
<28	165(78.2)	25(65.8)		158(78.2)	32(68.1)				
≥28	46(21.8)	13(34.2)		44(21.8)	15(31.9)				
Etiology of SAP, n(%)			0.769			0.674			
Biliary	124(49.4)	23(54.8)		116(48.5)	31(57.4)				
Hyperlipidemia	104(41.4)	14(33.3)		100(41.8)	18(33.3)				
Alcoholic	8(3.2)	2(4.8)		8(3.3)	2(3.7)				
Idiopathic	15(6.0)	3(7.1)		15(6.3)	3(5.6)				
Comorbidities, n(%)									
Hypertension	67(26.7)	16(38.1)	0.129	62(25.9)	21(38.9)	0.057			
DM	62(24.7)	7(16.7)	0.256	58(24.3)	11(20.4)	0.542			
Biliary tract disease	128(51.0)	17(40.5)	0.207	120(50.2)	25(46.3)	0.603			
Fatty liver	109(43.4)	20(47.6)	0.612	106(44.4)	23(42.6)	0.814			
Smoking, n(%)	98(39.0)	26(61.9)	0.006	92(38.5)	32(59.3)	0.005			
Drinking, n(%)	77(30.7)	24(57.1)	0.001	69(28.9)	32(59.3)	<0.001			

*HTBA* The high TBA group, *NTBA* The normal TBA group, *SD* Standard deviation, *BMI* Body mass index, *SAP* Severe acute pancreatitis, *DM* Diabetes mellitus

### Clinical outcomes

Results related to circulating TBA levels and prognosis of AP are shown in Table 2. There was a significant difference between the two groups in the severity of AP by DBC classification (All *P* < 0.05; Table 2). Most of patients in the HTBA group suffered from severe or critical AP, while in the NTBA groups mild and moderate AP were in the majority.

**Table 2:** Clinical outcomes of patients classified by TBA_max_

Variable	D1			D2			D3			D5			D7		
	NTBA	HTBA	*P* value	NTBA	HTBA	*P* value	NTBA	HTBA	*P* value	NTBA	HTBA	*P* value	NTBA	HTBA	*P* value
	*n* = 262	*n* = 17		*n* = 269	*n* = 22		*n* = 262	*n* = 31		*n* = 251	*n* = 42		*n* = 239	*n* = 54	
DBC classification, n(%)															
mild	111(42.4)	5(29.4)	0.002	113(42.0)	6(27.3)	0.001	109(41.6)	10(32.3)	0.033	109(43.4)	10(23.8)	<0.001	108(45.2)	11(20.4)	<0.001
moderate	109(41.6)	3(17.6)		113(42.0)	5(22.7)		109(41.6)	9(29.0)		106(42.2)	12(28.6)		105(43.9)	13(24.1)	
severe	30(11.5)	6(35.3)		30(11.2)	8(36.4)		31(11.8)	8(25.8)		25(10.0)	14(33.3)		20(8.4)	19(35.2)	
critical	12(4.6)	3(17.6)		13(4.8)	3(13.6)		13(5.0)	4(12.9)		11(4.4)	6(14.3)		6(2.5)	11(20.4)	
Organ failure, n(%)	58(22.1)	8(47.1)	0.04	61(22.7)	10(45.5)	0.017	60(22.9)	13(41.9)	0.02	51(20.3)	22(52.4)	<0.001	41(17.2)	32(59.3)	<0.001
ARDS, n(%)	46(17.6)	5(29.4)	0.367	48(17.8)	6(27.3)	0.419	48(18.3)	8(25.8)	0.316	42(16.7)	14(33.3)	0.011	33(13.8)	23(42.6)	<0.001
AKI, n(%)	37(14.1)	8(47.1)	0.001	38(14.1)	10(45.5)	<0.001	38(14.5)	12(38.7)	0.001	30(12.0)	20(47.6)	<0.001	22(9.2)	28(51.9)	<0.001
Shock, n(%)	18(6.9)	1(5.9)	1	18(6.7)	1(4.5)	1	19(7.3)	2(6.5)	1	16(6.4)	5(11.9)	0.336	11(4.6)	10(18.5)	0.001
Pancreatic necrosis, n(%)	140(53.4)	10(58.8)	0.666	145(53.9)	13(59.1)	0.639	142(54.2)	18(58.1)	0.683	131(52.2)	29(69.0)	0.042	121(50.6)	39(72.2)	0.004
PCD, n(%)	19 (7.3)	4 (23.5)	0.056	20 (7.4)	4 (18.2)	0.174	20 (7.6)	5 (16.1)	0.207	17 (6.8)	8 (19.0)	0.019	13(5.4)	12(22.2)	<0.001
Laparotomy, n(%)	7 (2.7)	0 (0.0)	1	8 (3.0)	0 (0.0)	1	8 (3.1)	0 (0.0)	1	7 (2.8)	1 (2.4)	1	5(2.1)	3(5.6)	0.343
Death, n(%)	9 (3.4)	2 (11.8)	0.139	9 (3.3)	2 (9.1)	0.198	9 (3.4)	3 (9.7)	0.238	7 (2.8)	5 (11.9)	0.019	4(1.7)	8(14.8)	<0.001

*HTBA* The high TBA group, *NTBA* The normal TBA group, *ARDS* Acute respiratory distress syndrome, *AKI* Acute kidney injury, *PCD* Percutaneous catheter drainage

Overall, organ failure developed in 73(24.9%) out of 293 patients were available. In these 73 patients, ARDS was the most common organ failure (76.7%), followed by AKI (68.5%) and Shock (28.8%). The incidence of organ failure was higher in the HTBA group than those in the NTBA group (All *P* < 0.05; Table 2). It is worth noting that in the HTBA group the incidence of AKI was higher than the NTBA group at each time point during the first 7 days after admission (All *P* < 0.01; Table 2), and this phenomenon was not observed in ARDS and shock (Table 2).

A total of 12 patients died, including 8 (14.8%) in the HTBA group and 4 (1.7%) in the NTBA group (Table 2). Most of the 12 deaths were caused by abdominal bleeding or septic shock due to infected pancreatic necrosis. In terms of (peri)pancreatic necrosis, PCD and mortality, there was no difference between the two groups in the first 5 days, and on the seventh day, the (peri)pancreatic necrosis rate, PCD rate and mortality of the HTBA group were significantly higher than those of the NTBA group (Table 2).

In order to eliminate the confounding effects of biliary pancreatitis, we compared the incidence of OF between the HTBA group and the NTBA group after excluding patients with biliary pancreatitis. It was found that the incidence of OF was still significantly higher in the HTBA group than the NTBA group (D7: 69.6% vs. 21.1%; *p* < 0.001). Similarly, the incidence of ARDS (D7: 43.5% vs. 15.4%; *p* = 0.005) and AKI (D7: 69.6% vs. 14.6%; *p* < 0.001) was also higher in the HTBA group. However, there was no significant difference in the incidence of shock between the two groups (D7: 17.4% vs. 7.3%; *p* = 0.247) (Additional file 1: Table S1).

### Univariate and multivariate analysis

In order to determine the potential risk factors for OF we applied univariate analysis and the result are shown in Additional file 2: Table S2. In the univariate analysis some clinical parameters involving inflammation, hepatobiliary diseases and OF were included (Additional file 2: Table S2). To adjust for baseline differences we incorporated age, gender, BMI ≥ 28 and some potentially confounding variables (*p* < 0.1 in the univariate analysis) into a multivariate model. As shown in Table 3, we found that HTBA (D7 TBA_max_ ≥ 10 μmol/L) was an independent risk factor for OF with an odds ratio of 4.894 (95% CI, 1.813–13.208; *p* = 0.002) (Table 3). However, at other time points (D1, D2, D3 and D5), the results shown that TBA_max_ ≥ 10 μmol/L was not an independent risk factor for OF (date not shown).

**Table 3:** Multivariate analysis showing association of proposed risk factors for organ failure in AP

Multivariate analysis	OR(95%CI)	*P* value
Age	1.009(0.974,1.044)	0.631
Male	0.967(0.321,2.913)	0.953
BMI ≥ 28	1.635(0.702,3.806)	0.254
Etiology		0.413
Biliary	2.022(0.298,13.716)	0.471
Hypertriglyceridemia	3.112(0.524,18.495)	0.212
Alcohol	0.550(0.016,18.537)	0.739
Biliary tract disease	0.481(0.170,1.361)	0.168
Fatty liver	1.588(0.693,3.638)	0.275
Smoking	1.474(0.567,3.834)	0.426
Drinking	0.728(0.305,1.740)	0.475
TBA_max_ ≥ 10 μmol/L	4.894(1.813,13.208)	0.002
TBIL	0.996(0.982,1.010)	0.595
AST	1.002(0.995,1.008)	0.58
WBC	1.064(0.986,1.148)	0.113
NEUT%	1.063(0.993,1.138)	0.08
CRP	1.002(0.996,1.007)	0.539
BUN	1.185(1.025,1.371)	0.022

*OR* Odds ratio, *CI* Confidence interval, *BMI* Body mass index, *TBAmax* The highest TBA value within 7 days after admission, *TBIL* Total bilirubin, *AST* Aspartate aminotransferase, *WBC* White blood cell count, *NEUT%* Neutrophil ratio, *CRP* C-reactive protein, *BUN* Blood urea nitrogen

To further validate our results, we performed a multivariate analysis after excluding biliary pancreatitis and the result are shown in Additional file 3: Table S3. HTBA (D7 TBA_max_ ≥ 10 μmol/L) was still an independent risk factor for OF with an odds ratio of 5.946 (95% CI, 1.043–33.894; *p* = 0.045) (Additional file 3: Table S3).

### Receiver operating characteristic curve analysis

**Fig. 2:**
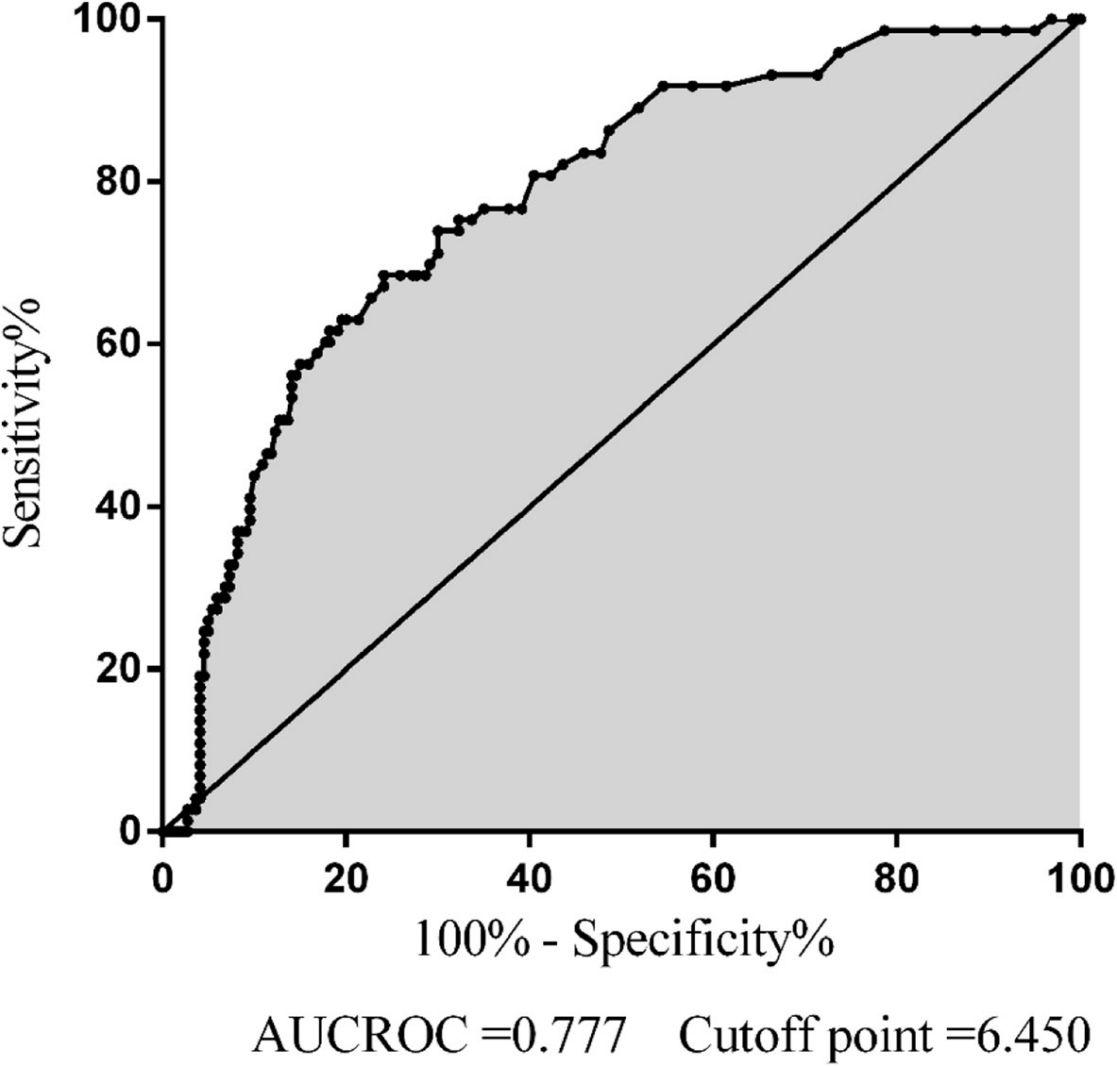
ROC analysis for the D7 TBA_max_ and the development of organ failure in acute pancreatitis. ROC, receiver operator characteristic; D7 TBA_max_, the maximum of serum TBA within 7 days after admission; AUCROC, area under the curve of the ROC curve

Receiver operator characteristic analysis revealed that a circulating D7 TBA_max_ cutoff point of 6.450 μmol/L had optimal predictive value for the development of OF in AP patients. The sensitivity of the cutoff point was 68.5% and the specificity was 75.9% with an AUCROC of 0.777, which was greater than BUN (Fig. 2). Moreover, we used the D7 TBA_max_ cutoff point for verification, and found that patients with D7 TBA_max_ exceeding 6.450 umol/L did have a higher rate of OF (Additional file 4: Table S4). Besides, HTBA (D7 TBA_max_ ≥ 6.450 μmol/L) was still an independent risk factor for OF with an odds ratio of 6.261 (95% CI, 2.835–13.830; *p* < 0.001) (Additional file 5: Table S5).

## Discussion

Organ failure and (peri)pancreatic necrosis are two key factors that are causally associated with the severity of AP [21]. A large number of investigations demonstrated a statistically significant association between a wide array of factors and the severity of acute pancreatitis, such as prolonged hospitalization and need for intervention, but the relationships are non-causal [22, 23]. Therefore, the early and accurate prediction of organ failure is particularly important in the treatment of SAP patients. The widely adopted guidelines of the International Association of Pancreatology and the American Pancreatic Association recommend the persistent (lasting≥48 h) SIRS as early markers to predict the development of organ failure [24]. However, despite a reasonably good sensitivity of 50–95%, SIRS has a lower specificity of 75% [25, 26]. Several clinical scores can also be exploited to predict the development of organ failure in AP, such as APACHE II, BISAP score and SOFA score [27], which are involved multiple parameters and are somewhat cumbersome to use. Single laboratory markers (including IL-6, CRP, and procalcitonin) can also be used as a sensitive marker, but the guidelines emphasize the need for a repeated clinical assessment [24, 28, 29]. At present, no single laboratory marker can be recommended for the early prediction of the development of OF in AP. Recent studies showed that angiopoietin-2(a marker of vascular leak syndrome) [30] or serum urokinase-type plasminogen activator receptor (uPAR) [31] can be applied as a marker in predicting persistent organ failure. Unfortunately, the detections of these indicators are not widely used so far and is difficult to obtain in clinical practice. It is necessary to find early and appropriate markers of organ failure, which can help doctors identify critically ill patients and allow for the proper allocation of intensive care resources in time.

BAs are physiological detergent molecules synthesized from cholesterol in the liver. Dietary intake stimulates Bile acids into the intestines and then they can facilitate the absorption of dietary lipids and vitamins in the intestines [32].Over the past few decades, many studies have suggested that bile acids are signalling molecules that regulate lipid, glucose and energy metabolism which are predominately mediated by bile acid-activated FXR and TGR5 [8, 9, 14, 33]. When the homeostasis of BAs is broken or the signaling pathway is impaired, it can lead to a variety of metabolic disorders or inflammatory diseases [14, 33]. At present, circulating TBA can be used as a marker for diagnosing hepatobiliary diseases, and has been widely used in clinical work [16, 34]. Besides, some studies have reported that circulating TBA can predict the occurrence of colorectal cancer [35, 36] and pancreatic cancer [37]. However, there are few researches in the field of critical illness or AP involving BAs.

The results of our study shown that 54 (18.43%) patients with AP had a D7 TBA_max_ ≥ 10 μmol/L within 7 days after admission, and the increase of circulating TBA was not only observed in biliary AP, but also in hypertriglyceridemic and alcoholic AP. Further research revealed that the incidence of OF in the HTBA group was significantly higher than that in the NTBA group, and the AP severity classification in the HTBA group was more serious than that in the NTBA group. We performed a stratified analysis at multiple time points, and in addition we repeated the comparative analysis after biliary AP was excluded, thereby further verifying the reliability of the results. The pancreatic necrosis rate, PCD rate and mortality in the HTBA group were higher than those in the NTBA group according to the D7 TBA_max_ values. All of the above results indicated that the increase in circulating TBA in AP patients was not related to the etiology, and the AP patients with HTBA had a worse prognosis.

The pathophysiological mechanism of AP complicated with OF is that pancreatitis per se (sterile inflammation) causes the release of a large number of inflammatory mediators leading to primary (early) OF or infected pancreatic necrosis leads to the secondary (late) OF [27]. Cholestasis is a common complication of sepsis. Hao et al. showed that the amplified plasma levels of BAs are important for the prediction of sepsis-associated mortality. They proved that bile acids activate the NLRP3 inflammasome via promoting calcium influx. Their research also depicted that the FXR-bile acid axis involves in the regulation of cholestasis-associated sepsis, which can be mediated by the negative regulation of NLRP3 inflammasome via the direct binding of FXR to NLRP3 and caspase 1 in macrophages [38, 39]. Maleszka et al. reported that circulating TBA on the first day of AP could be used to discriminate the biliary AP from the other etiologies. The cut-off values of 4.7 mol/L, with a diagnostic accuracy of 85%. However, circulating TBA had nothing to do with the severity of AP [15]. On the contrary, in this present study, we found that the levels of circulating TBA were closely related to the severity of AP, and logistic analysis suggested that circulating D7 HTBA_max_ was an independent risk factor for AP complicated with organ failure. Our results are somewhat different from those of Maleszka et al. The reasons for the differences may be in the following aspects: first, in their study, the values of circulating TBA were continuously monitored for 3 days after AP onset, while the time span of our test was longer; secondly, the sample sizes of two groups were relatively small in our study, which is prone to bias; finally, our center is a SAP tertiary referral center in which our patients are more serious.

In recent years, the pathophysiological mechanisms of BAs and its receptors in diseases were elucidated in some researches. Iracheta-Vellve A et al. manifested that agonists of FXR and TGR5 (OCA, INT-767 and INT-777) can reduce the expression of inflammatory cytokine in animal models of alcoholic liver disease by inhibiting macrophage inflammation through activation of protein kinase A induced by cyclic adenosine monophosphate (cAMP) [40]. This mechanism was confirmed in TGR5 ligand ameliorating the immunity of intestinal mucosa in experimental colitis [41]. Moreover, in the pancreas, local accumulation of BAs molecules could inhibit autophagy of pancreatic acinar cells through FXR, leading to the increasing of apoptosis and necrotic apoptosis [42]. Our team previously found that the administration of INT-777 could protect AP in mice and improve pancreatic acinar cell necrosis [43]. Based on the above studies, we hypothesize that the imbalance of BAs, which may lead to the disorder in BAs metabolism and inflammatory response, thus affecting the organ function. Especially after a certain period of standardized treatment, the circulating TBA levels are still higher than normal during the observation period, which deserves our attention. However, the specific mechanism of increased circulating TBA levels associated with organ failure remains unclear which needs further study.

Our study reports for the first time that circulating TBA levels in the early stage of AP patients are associated with the development of organ failure. This can help clinicians identify patients whom are at risk of organ failure in the early stage of AP (within 14 days), so as to treat them promptly and reduce the mortality rate. In addition, the detection of circulating TBA has been widely used, exerting multiple effects of the same indicator. However, our research also has some limitations. First, this is a single-center retrospective study with a small sample size and further research with larger sample sizes is needed. Moreover, our observation marker, TBA_max_, was the highest value of circulating TBA within 7 days of admission. Long monitoring time span may cause some bias to the results. Finally, our study cannot completely rule out the effects of liver injury and drug application on circulating TBA.

## Conclusions

Overall, elevation of circulating TBA in the early stage of AP is independently associated with organ failure, which indicates the adverse prognosis of AP patients.

## Supplementary information

**Additional file 1 Table S1.** Comparison of the incidence of organ failure after biliary pancreatitis were excluded. HTBA, the high TBA group; NTBA, the normal TBA group; ARDS, acute respiratory distress syndrome; AKI, acute kidney injury.

**Additional file 2 Table S2.** Univariate analysis showing the potential risk factors for organ failure in acute pancreatitis. OR, Odds ratio; CI, confidence interval; BMI, body mass index; DM, diabetes mellitus; TBA, total bile acid; TBIL, total bilirubin; ALP, alkaline phosphatase; γ-GT, γ-glutamyl transpeptadase; ALT, alanine aminotransferase; AST, aspartate aminotransferase; WBC, white blood cell count; NEUT%, neutrophil ratio; CRP, C-reactive protein; PLT, platelet; BUN, blood urea nitrogen.

**Additional file 3 Table S3.** Multivariate analysis showing association of risk factors for OF after biliary pancreatitis were excluded. OR, Odds ratio; CI, confidence interval; BMI, body mass index; TBA_max_, the highest TBA value within 7 days after admission; TBIL, total bilirubin; AST, aspartate aminotransferase; WBC, white blood cell count; NEUT%, neutrophil ratio; CRP, C-reactive protein; BUN, blood urea nitrogen.

**Additional file 4 Table S4.** Clinical outcomes of patients classified by TBA_max_ cutoff point. HTBA, the high TBA group; NTBA, the normal TBA group; ARDS, acute respiratory distress syndrome; AKI, acute kidney injury; PCD, percutaneous catheter drainage.

**Additional file 5 Table S5.** Multivariate regression analysis verifying the accuracy of TBA_max_ cutoff point. OR, Odds ratio; CI, confidence interval; BMI, body mass index; TBA_max_, the highest TBA value within 7 days after admission; TBIL, total bilirubin; AST, aspartate aminotransferase; WBC, white blood cell count; NEUT%, neutrophil ratio; CRP, C-reactive protein; BUN, blood urea nitrogen.

## Data Availability

The datasets analysed during the current study are available from the corresponding author on reasonable request.

## Abbreviations

BAs: Bile acids
TBA: Total bile acid
AP: Acute pancreatitis
HTBA: High TBA
NTBA: Normal TBA
OF: Organ failure
PCD: Percutaneous catheter drainage
OR: Odds ratio
SAP: Severe acute pancreatitis
ARDS: Acute respiratory distress syndrome
AKI: Acute kidney injury
FXR: Farnesoid X receptor
RRT: Renal replacement therapy
UDCA: Ursodeoxycholic acid
PBC: Primary biliary cholangitis
PSC: Primary sclerosing cholangitis
SD: Standard deviation
IQR: Interquartile range
BMI: Body mass index
DM: Diabetes mellitus
CI: Confidence interval
TBIL: Total bilirubin
ALP: Alkaline phosphatase
γ-GT: γ-glutamyl transpeptadase
ALT: Alanine aminotransferase
AST: Aspartate aminotransferase
WBC: White blood cell count
NEUT%: Neutrophil ratio
CRP: C-reactive protein
PLT: Platelet
BUN: Blood urea nitrogen

## References

[1] Yadav D, Lowenfels AB (2013). The epidemiology of pancreatitis and pancreatic cancer. Gastroenterology..

[2] Peery AF, Dellon ES, Lund J, Crockett SD, McGowan CE, Bulsiewicz WJ (2012). Burden of gastrointestinal disease in the United States: 2012 update. Gastroenterology.

[3] Tenner S, Baillie J, DeWitt J, Vege SS (2013). American College of Gastroenterology guideline: management of acute pancreatitis. Am J Gastroenterol.

[4] Banks PA, Freeman ML (2006). Practice guidelines in acute pancreatitis. Am J Gastroenterol.

[5] Babu RY, Gupta R, Kang M, Bhasin DK, Rana SS, Singh R (2013). Predictors of surgery in patients with severe acute pancreatitis managed by the step-up approach. Ann Surg.

[6] Banks PA, Bollen TL, Dervenis C, Gooszen HG, Johnson CD, Sarr MG (2013). Classification of acute pancreatitis--2012: revision of the Atlanta classification and definitions by international consensus. Gut..

[7] Li T, Chiang JY (2014). Bile acid signaling in metabolic disease and drug therapy. Pharmacol Rev.

[8] Fuchs C, Claudel T, Trauner M (2013). Bile acid-mediated control of liver triglycerides. Semin Liver Dis.

[9] Li T, Chiang JY (2015). Bile acids as metabolic regulators. Curr Opin Gastroenterol.

[10] McGlone ER, Bloom SR. ANNALS EXPRESS: Bile acids and the metabolic syndrome. Ann Clin Biochem. 2019;56(3):326–37. 10.1177/0004563218817798 30453753

[11] Han CY. Update on FXR Biology: Promising Therapeutic Target? Int J Mol Sci. 2018;19(7):2069. 10.3390/ijms19072069 PMC6073382 30013008

[12] Xie Y, He Y, Cai Z, Cai J, Xi M, Zhang Y (2016). Tauroursodeoxycholic acid inhibits endoplasmic reticulum stress, blocks mitochondrial permeability transition pore opening, and suppresses reperfusion injury through GSK-3ss in cardiac H9c2 cells. Am J Transl Res.

[13] Cummings BP, Bettaieb A, Graham JL, Kim J, Ma F, Shibata N (2013). Bile-acid-mediated decrease in endoplasmic reticulum stress: a potential contributor to the metabolic benefits of ileal interposition surgery in UCD-T2DM rats. Dis Model Mech.

[14] Trauner M, Claudel T, Fickert P, Moustafa T, Wagner M (2010). Bile acids as regulators of hepatic lipid and glucose metabolism. Dig Dis.

[15] Maleszka A, Dumnicka P, Matuszyk A, Pedziwiatr M, Mazur-Laskowska M, Sporek M, et al. The Diagnostic Usefulness of Serum Total Bile Acid Concentrations in the Early Phase of Acute Pancreatitis of Varied Etiologies. Int J Mol Sci. 2017;18(1):106. 10.3390/ijms18010106 PMC5297740 28067818

[16] Bouchier IA, Pennington CR (1978). Serum bile acids in hepatobiliary disease. Gut..

[17] Haeusler RA, Camastra S, Nannipieri M, Astiarraga B, Castro-Perez J, Xie D (2016). Increased bile acid synthesis and impaired bile acid transport in human obesity. J Clin Endocrinol Metab.

[18] Sonne DP, van Nierop FS, Kulik W, Soeters MR, Vilsboll T, Knop FK (2016). Postprandial plasma concentrations of individual bile acids and FGF-19 in patients with type 2 diabetes. J Clin Endocrinol Metab.

[19] Wewalka M, Patti ME, Barbato C, Houten SM, Goldfine AB (2014). Fasting serum taurine-conjugated bile acids are elevated in type 2 diabetes and do not change with intensification of insulin. J Clin Endocrinol Metab.

[20] Sun W, Zhang D, Wang Z, Sun J, Xu B, Chen Y (2016). Insulin resistance is associated with Total bile acid level in type 2 diabetic and nondiabetic population: a cross-sectional study. Medicine..

[21] Dellinger EP, Forsmark CE, Layer P, Levy P, Maravi-Poma E, Petrov MS (2012). Determinant-based classification of acute pancreatitis severity: an international multidisciplinary consultation. Ann Surg.

[22] Petrov MS, Windsor JA (2012). Conceptual framework for classifying the severity of acute pancreatitis. Clin Res Hepatol Gastroenterol.

[23] Hennekens CH, DeMets D (2011). Statistical association and causation: contributions of different types of evidence. Jama..

[24] Working Group IAP/APA Acute Pancreatitis Guidelines (2013). IAP/APA evidence-based guidelines for the management of acute pancreatitis. Pancreatology.

[25] Mofidi R, Duff MD, Wigmore SJ, Madhavan KK, Garden OJ, Parks RW (2006). Association between early systemic inflammatory response, severity of multiorgan dysfunction and death in acute pancreatitis. Br J Surg.

[26] Singh VK, Wu BU, Bollen TL, Repas K, Maurer R, Mortele KJ (2009). Early systemic inflammatory response syndrome is associated with severe acute pancreatitis. Clin Gastroenterol Hepatol.

[27] Garg PK, Singh VP (2019). Organ Failure due to Systemic Injury in Acute Pancreatitis. Gastroenterol.

[28] Parniczky A, Kui B, Szentesi A, Balazs A, Szucs A, Mosztbacher D (2016). Prospective, multicentre, Nationwide clinical data from 600 cases of acute pancreatitis. PLoS One.

[29] Sathyanarayan G, Garg PK, Prasad H, Tandon RK (2007). Elevated level of interleukin-6 predicts organ failure and severe disease in patients with acute pancreatitis. J Gastroenterol Hepatol.

[30] Whitcomb DC, Muddana V, Langmead CJ, Houghton FD, Guenther A, Eagon PK (2010). Angiopoietin-2, a regulator of vascular permeability in inflammation, is associated with persistent organ failure in patients with acute pancreatitis from the United States and Germany. Am J Gastroenterol.

[31] Kolber W, Kusnierz-Cabala B, Dumnicka P, Maraj M, Mazur-Laskowska M, Pedziwiatr M, et al. Serum Urokinase-Type Plasminogen Activator Receptor Does Not Outperform C-Reactive Protein and Procalcitonin as an Early Marker of Severity of Acute Pancreatitis. J Clin Med. 2018;7(10):305. 10.3390/jcm7100305 PMC6210514 30262764

[32] Chiang JY (2002). Bile acid regulation of gene expression: roles of nuclear hormone receptors. Endocr Rev.

[33] Chiang JYL (2017). Bile acid metabolism and signaling in liver disease and therapy. Liver Res.

[34] Luo L, Aubrecht J, Li D, Warner RL, Johnson KJ, Kenny J (2018). Assessment of serum bile acid profiles as biomarkers of liver injury and liver disease in humans. PLoS One.

[35] Bayerdorffer E, Mannes GA, Ochsenkuhn T, Dirschedl P, Wiebecke B, Paumgartner G (1995). Unconjugated secondary bile acids in the serum of patients with colorectal adenomas. Gut..

[36] Costarelli V, Key TJ, Appleby PN, Allen DS, Fentiman IS, Sanders TA (2002). A prospective study of serum bile acid concentrations and colorectal cancer risk in post-menopausal women on the island of Guernsey. Br J Cancer.

[37] Feng HY, Chen YC (2016). Role of bile acids in carcinogenesis of pancreatic cancer: an old topic with new perspective. World J Gastroenterol.

[38] Hao H, Cao L, Jiang C, Che Y, Zhang S, Takahashi S (2017). Farnesoid X Receptor Regulation of the NLRP3 Inflammasome Underlies Cholestasis-Associated Sepsis. Cell Metab.

[39] Garcia-Irigoyen O, Moschetta A (2017). A novel protective role for FXR against Inflammasome activation and Endotoxemia. Cell Metab.

[40] Iracheta-Vellve A, Calenda CD, Petrasek J, Ambade A, Kodys K, Adorini L (2018). FXR and TGR5 agonists ameliorate liver injury, Steatosis, and inflammation after binge or prolonged alcohol feeding in mice. Hepatol Commun.

[41] Cipriani S, Mencarelli A, Chini MG, Distrutti E, Renga B, Bifulco G (2011). The bile acid receptor GPBAR-1 (TGR5) modulates integrity of intestinal barrier and immune response to experimental colitis. PLoS One.

[42] Zhou X, Xie L, Bergmann F, Endris V, Strobel O, Buchler MW (2017). The bile acid receptor FXR attenuates acinar cell autophagy in chronic pancreatitis. Cell Death Dis.

[43] Li B, Yang N, Li C, Li C, Gao K, Xie X (2018). INT-777, a bile acid receptor agonist, extenuates pancreatic acinar cells necrosis in a mouse model of acute pancreatitis. Biochem Biophys Res Commun.

