# Supplementary material for: Increased circulating total bile acid levels were associated with organ failure in patients with acute pancreatitis
Source: BMC Gastroenterol. 2020 Jul 13;20:222. doi: 10.1186/s12876-020-01243-w (PMC7359019; doi:10.1186/s12876-020-01243-w)
Supplement: Supplementary file 1 — Additional file 1 Table S1. Comparison of the incidence of organ failure after biliary pancreatitis were excluded. HTBA, the high TBA group; NTBA, the normal TBA group; ARDS, acute respiratory distress syndrome; AKI, acute kidney injury. [file 12876_2020_1243_MOESM1_ESM.docx]

| Variable | | | Organ failure, n(%) | ARDS, n(%) | AKI, n(%) | Shock, n(%) |
| --- | --- | --- | --- | --- | --- | --- |
| D1 | NTBA | n=136 | 36（26.5） | 25（18.4） | 28（20.6） | 12（8.8） |
|  | HTBA | n=5 | 3（60.0） | 2（40.0） | 3（60.0） | 0（00.0） |
|  | *P* value | | 0.255 | 0.244 | 0.124 | 1 |
| D2 | NTBA | n=138 | 37（26.8） | 25（18.1） | 29（21.0） | 12（8.7） |
|  | HTBA | n=7 | 4（57.1） | 3（42.9） | 4（57.1） | 0（00.0） |
|  | *P* value | | 0.191 | 0.26 | 0.078 | 1 |
| D3 | NTBA | n=135 | 36（26.7） | 25（18.5） | 28（20.7） | 12（8.9） |
|  | HTBA | n=11 | 6（54.5） | 4（36.4） | 6（54.5） | 1（9.1） |
|  | *P* value | | 0.106 | 0.301 | 0.029 | 1 |
| D5 | NTBA | n=127 | 30（23.6） | 22（17.3） | 22（17.3） | 11（8.7） |
|  | HTBA | n=19 | 12（63.2） | 7（36.8） | 12（63.2） | 2（10.5） |
|  | *P* value | | <0.001 | 0.093 | <0.001 | 1 |
| D7 | NTBA | n=123 | 26（21.1） | 19（15.4） | 18（14.6） | 9（7.3） |
|  | HTBA | n=23 | 16（69.6） | 10（43.5） | 16（69.6） | 4（17.4） |
|  | *P* value | | <0.001 | 0.005 | <0.001 | 0.247 |
