# Supplementary material for: Increased circulating total bile acid levels were associated with organ failure in patients with acute pancreatitis
Source: BMC Gastroenterol. 2020 Jul 13;20:222. doi: 10.1186/s12876-020-01243-w (PMC7359019; doi:10.1186/s12876-020-01243-w)
Supplement: Supplementary file 2 — Additional file 2 Table S2. Univariate analysis showing the potential risk factors for organ failure in acute pancreatitis. OR, Odds ratio; CI, confidence interval; BMI, body mass index; DM, diabetes mellitus; TBA, total bile acid; TBIL, total bilirubin; ALP, alkaline phosphatase; γ-GT, γ-glutamyl transpeptadase; ALT, alanine aminotransferase; AST, aspartate aminotransferase; WBC, white blood cell count; NEUT%, neutrophil ratio; CRP, C-reactive protein; PLT, platelet; BUN, blood urea nitrogen. [file 12876_2020_1243_MOESM2_ESM.docx]

| Univariate analysis | OR(95%CI) | *P* value |
| --- | --- | --- |
| Age | 0.997(0.976,1.018) | 0.786 |
| Male | 1.887(1.038,3.431) | 0.037 |
| BMI≥28 | 2.396(1.278,4.493) | 0.006 |
| Etiology |  | 0.047 |
| Biliary | 2.138(0.466,9.799) | 0.328 |
| Hypertriglyceridemia | 3.949(0.864,18.043) | 0.076 |
| Alcohol | 0.889(0.070,11.221) | 0.927 |
| Hypertension | 1.455(0.824,2.510) | 0.197 |
| DM | 0.795(0.417,1.516) | 0.486 |
| Biliary tract disease | 0.591(0.345,1.012) | 0.055 |
| Fatty liver | 2.233(1.303,3.829) | 0.003 |
| Smoking | 2.280(1.331,3.907) | 0.003 |
| Drinking | 1.844(1.072,3.172) | 0.027 |
| TBA | 7.024(3.709,13.302) | ＜0.001 |
| TBIL | 1.018(1.008,1.027) | ＜0.001 |
| ALP | 1.003(0.999,1.007) | 0.138 |
| r-GT | 1.000(0.998,1.001) | 0.986 |
| ALT | 1.001(0.999,1.003) | 0.411 |
| AST | 1.006(1.002,1.011) | 0.005 |
| WBC | 1.107(1.056,1.161) | ＜0.001 |
| NEUT% | 1.113(1.065,1.163) | ＜0.001 |
| CRP | 1.006(1.003,1.010) | 0.001 |
| PLT | 0.997(0.993,1.001) | 0.131 |
| BUN | 1.025(1.014,1.035) | ＜0.001 |
