# Supplementary material for: Increased circulating total bile acid levels were associated with organ failure in patients with acute pancreatitis
Source: BMC Gastroenterol. 2020 Jul 13;20:222. doi: 10.1186/s12876-020-01243-w (PMC7359019; doi:10.1186/s12876-020-01243-w)
Supplement: Supplementary file 3 — Additional file 3 Table S3. Multivariate analysis showing association of risk factors for OF after biliary pancreatitis were excluded. OR, Odds ratio; CI, confidence interval; BMI, body mass index; TBAmax, the highest TBA value within 7 days after admission; TBIL, total bilirubin; AST, aspartate aminotransferase; WBC, white blood cell count; NEUT%, neutrophil ratio; CRP, C-reactive protein; BUN, blood urea nitrogen. [file 12876_2020_1243_MOESM3_ESM.docx]

| Multivariate analysis | OR(95%CI） | P value |
| --- | --- | --- |
| Age | 0.988(0.938,1.039) | 0.631 |
| Male | 0.524(0.095,2.883) | 0.458 |
| BMI≥28 | 1.183(0.399,3.507) | 0.761 |
| Etiology |  | 0.148 |
| Hypertriglyceridemia | 7.000(0.854,57.383) | 0.07 |
| Alcohol | 1.218(0.026,56.927) | 0.92 |
| Biliary tract disease | 0.791(0.169,3.710) | 0.766 |
| Fatty liver | 0.555(0.168,1.830) | 0.333 |
| Smoking | 2.671(0.563,12.680) | 0.216 |
| Drinking | 0.679(0.198,2.327) | 0.538 |
| TBA_max_≥10μmol/L | 5.946(1.043,33.894) | 0.045 |
| TBIL | 0.994(0.974,1.015) | 0.579 |
| AST | 1.003(0.993,1.013) | 0.521 |
| WBC | 1.085(0.962,1.223) | 0.186 |
| NEUT% | 1.034(0.944,1.134) | 0.47 |
| CRP | 1.001(0.993,1.009) | 0.825 |
| BUN | 1.330(1.062,1.666) | 0.013 |
