# Supplementary material for: Increased circulating total bile acid levels were associated with organ failure in patients with acute pancreatitis
Source: BMC Gastroenterol. 2020 Jul 13;20:222. doi: 10.1186/s12876-020-01243-w (PMC7359019; doi:10.1186/s12876-020-01243-w)
Supplement: Supplementary file 4 — Additional file 4 Table S4. Clinical outcomes of patients classified by TBAmax cutoff point. HTBA, the high TBA group; NTBA, the normal TBA group; ARDS, acute respiratory distress syndrome; AKI, acute kidney injury; PCD, percutaneous catheter drainage. [file 12876_2020_1243_MOESM4_ESM.docx]

| Variable | NTBA | HTBA | *P* value |
| --- | --- | --- | --- |
|  | n=190 | n=103 |  |
| DBC classification, n(%) |  |  |  |
| mild | 94（49.5） | 25（24.3） | ＜0.001 |
| moderate | 81（42.6） | 37（35.9） |  |
| severe | 13（6.8） | 26（25.2） |  |
| critical | 2（1.1） | 15（14.6） |  |
| Organ failure, n(%) | 23（12.1） | 50（48.5） | ＜0.001 |
| ARDS, n(%) | 18（9.5） | 38（36.9） | ＜0.001 |
| AKI, n(%) | 11（5.8） | 39（37.9） | ＜0.001 |
| Shock, n(%) | 5（2.6） | 16（15.5） | ＜0.001 |
| Pancreatic necrosis, n(%) | 91（47.9） | 69（67.0） | 0.002 |
| PCD, n(%) | 8 (4.2) | 17 (16.5) | ＜0.001 |
| Laparotomy, n(%) | 1 (0.5) | 7 (6.8) | 0.006 |
| Death, n(%) | 2 (1.1) | 10 (9.7) | 0.001 |
