# Supplementary material for: Increased circulating total bile acid levels were associated with organ failure in patients with acute pancreatitis
Source: BMC Gastroenterol. 2020 Jul 13;20:222. doi: 10.1186/s12876-020-01243-w (PMC7359019; doi:10.1186/s12876-020-01243-w)
Supplement: Supplementary file 5 — Additional file 5 Table S5. Multivariate regression analysis verifying the accuracy of TBAmax cutoff point. OR, Odds ratio; CI, confidence interval; BMI, body mass index; TBAmax, the highest TBA value within 7 days after admission; TBIL, total bilirubin; AST, aspartate aminotransferase; WBC, white blood cell count; NEUT%, neutrophil ratio; CRP, C-reactive protein; BUN, blood urea nitrogen. [file 12876_2020_1243_MOESM5_ESM.docx]

| Multivariate analysis | OR(95%CI） | *P* value |
| --- | --- | --- |
| Age | 1.013(0.976,1.052) | 0.486 |
| Male | 1.199(0.382,3.764) | 0.756 |
| BMI≥28 | 1.381(0.570,3.346) | 0.475 |
| Etiology |  | 0.414 |
| Biliary | 1.314(0.189,9.122) | 0.782 |
| Hypertriglyceridemia | 2.163(0.362,12.91) | 0.397 |
| Alcohol | 0.274(0.009,8.583) | 0.461 |
| Biliary tract disease | 0.66(0.222,1.967) | 0.456 |
| Fatty liver | 1.512(0.647,3.533) | 0.34 |
| Smoking | 1.565(0.582,4.21) | 0.375 |
| Drinking | 0.771(0.309,1.923) | 0.577 |
| TBA_max_≥6.450μmol/L | 6.261(2.835,13.83) | <0.001 |
| TBIL | 0.997(0.983,1.011) | 0.704 |
| AST | 1.002(0.995,1.008) | 0.592 |
| WBC | 1.079(0.995,1.171) | 0.065 |
| NEUT% | 1.059(0.987,1.137) | 0.111 |
| CRP | 1.002(0.996,1.008) | 0.464 |
| BUN | 1.212(1.04,1.413) | 0.014 |
